# Hydroxy-Propil-β-Cyclodextrin Inclusion Complexes of two Biphenylnicotinamide Derivatives: Formulation and Anti-Proliferative Activity Evaluation in Pancreatic Cancer Cell Models

**DOI:** 10.3390/ijms21186545

**Published:** 2020-09-07

**Authors:** Rosa Maria Iacobazzi, Annalisa Cutrignelli, Angela Stefanachi, Letizia Porcelli, Angela Assunta Lopedota, Roberta Di Fonte, Antonio Lopalco, Simona Serratì, Valentino Laquintana, Nicola Silvestris, Massimo Franco, Saverio Cellamare, Francesco Leonetti, Amalia Azzariti, Nunzio Denora

**Affiliations:** 1 Laboratory of Experimental Pharmacology, IRCCS Istituto Tumori Giovanni Paolo II, 70124 Bari, Italy; rosamaria.iacobazzi@gmail.com (R.M.I.); porcelli.letizia@gmail.com (L.P.); difonte.roberta@gmail.com (R.D.F.); n.silvestris@oncologico.bari.it (N.S.); a.azzariti@oncologico.bari.it (A.A.); 2 Department of Pharmacy-Drug Sciences, University of Bari “Aldo Moro”, 70125 Bari, Italy; annalisa.cutrignelli@uniba.it (A.C.); angela.stefanachi@uniba.it (A.S.); angelaassunta.lopedota@uniba.it (A.A.L.); antonio.lopalco@uniba.it (A.L.); valentino.laquintana@uniba.it (V.L.); massimo.franco@uniba.it (M.F.); saverio.cellamare@uniba.it (S.C.); francesco.leonetti@uniba.it (F.L.); 3 Laboratory of Nanotechnology, IRCCS Istituto Tumori Giovanni Paolo II, 70124 Bari, Italy; simonaserrati@hotmail.com; 4 Department of Biomedical Sciences and Human Oncology, University of Bari “Aldo Moro”, 70124 Bari, Italy

**Keywords:** pancreatic ductal adenocarcinoma, cyclodextrin inclusion complex, phase solubility studies, preformulation studies, biphenylnicotinamide derivatives

## Abstract

Pancreatic ductal adenocarcinoma (PDAC) is one of the most aggressive malignancies, with poor outcomes largely due to its unique microenvironment, which is responsible for the low response to drugs and drug-resistance phenomena. This clinical need led us to explore new therapeutic approaches for systemic PDAC treatment by the utilization of two newly synthesized biphenylnicotinamide derivatives, PTA73 and PTA34, with remarkable antitumor activity in an in vitro PDAC model. Given their poor water solubility, inclusion complexes of PTA34 and PTA73 in Hydroxy-Propil-β-Cyclodextrin (HP-β-CD) were prepared in solution and at the solid state. Complexation studies demonstrated that HP-β-CD is able to form stable host–guest inclusion complexes with PTA34 and PTA73, characterized by a 1:1 apparent formation constant of 503.9 M^−1^ and 369.2 M^−1^, respectively (also demonstrated by the Job plot), and by an increase in aqueous solubility of about 150 times (from 1.95 µg/mL to 292.5 µg/mL) and 106 times (from 7.16 µg/mL to 762.5 µg/mL), in the presence of 45% *w/v* of HP-β-CD, respectively. In vitro studies confirmed the high antitumor activity of the complexed PTA34 and PTA73 towards PDAC cells, the strong G2/M phase arrest followed by induction of apoptosis, and thus their eligibility for PDAC therapy.

## 1. Introduction

Pancreatic ductal adenocarcinoma (PDAC) is the most common type of pancreatic cancer, which kills more patients every year than any other type of cancer excluding lung and colorectal. Although accounting for only 3% of new cancer cases in the United States, it is responsible for over 7% of all cancer deaths, with an overall five-year survival of less than 5% [1]. In 2019, in Italy, 13,500 new cases were expected (6800 in men and 6700 in women), about 3% of all male and female cancers [2]. The American Cancer Society estimates that in 2020 about 57,600 people (30,400 men and 27,200 women) will be diagnosed with pancreatic cancer, and that about 47,050 people (24,640 men and 22,410 women) will die of pancreatic cancer [1].

Since PDAC is generally diagnosed at an advanced stage, systemic therapy is the main strategy of treatment. Currently, the most successful chemotherapy regimens for this type of tumor are gemcitabine, FOLFIRINOX, and the combination gemcitabine/nabpaclitaxel. However, the clinical management of patients still remains an open challenge, because in most cases patients have inherent resistance to therapies. The poor outcome for PDAC patients is mainly due to the peculiarity of the desmoplastic stroma that represents up to 90% of the tumor mass, and is characterized by fibrosis, poor vascularization, high intratumoral pressure, immune infiltrates, and hypoxia, with consequent reduction of the bioavailability of the drugs also hindered by rapid elimination, metabolic inactivation, and not specific systemic toxicity [3,4].

The interaction between pancreatic cancer cells and the tumor microenvironment, including immune cells, endothelial cells, and fibroblasts, plays a crucial role in PDAC development and progression and in drug-resistance phenomena [5].

**Figure 1:**
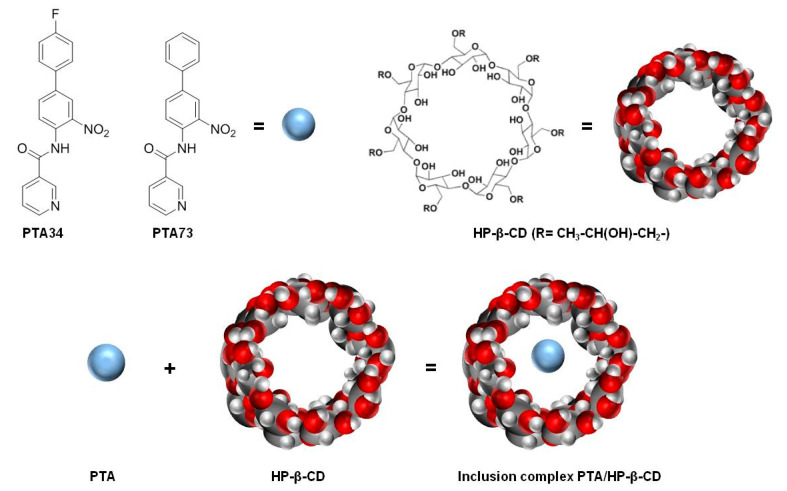
Chemical structures of PTA34, PTA73, hydroxy-propil-β-cyclodextrin (HP-β-CD) and graphical representation of the inclusion complex.

In this scenario, in order to identify new therapeutic strategies for PDAC, we planned to investigate, in a PDAC cells panel, the pharmacological efficacy of two newly synthesized N-biphenylnicotinamides, namely PTA34 and PTA73 [6,7], formulated as hydroxy-propil-β-cyclodextrin (HP-β-CD) inclusion complexes (Figure 1).

PTA34 and PTA73 molecules have been already classified as a novel, highly potent, and selective class of microtubule targeting agents (MTAs) and potential anti-angiogenic and vascular-disrupting agents in the Hodgkin lymphoma model [7]. Moreover, a remarkable antitumor activity of these molecules at low doses was assessed also in a PDAC model, MIA PaCa-2 cells [7], confirming that targeting microtubule dynamics could be effective against the abnormal proliferation of PDAC cancer cells [8,9,10].

However, preformulation studies conducted on PTA34 and PTA73 showed very low water solubility, which strongly limits the potential pharmaceutical development for these compounds due to the poor bioavailability of the drug. Therefore, the improvement of the aqueous solubility for the new PTA’s formulations was an urgent need.

Different formulation strategies allow overcoming the limits of poorly soluble drugs, such as solid dispersions [11,12], addition of cosolvents [13], complexation, and size reduction [14,15], however the most studied and applied approach to improve the solubility and bioavailability of drugs is the complexation in cyclodextrins [16,17,18].

Cyclodextrins (CDs), are cyclic oligosaccharides containing at least 6 D - (+) glucopyranose units attached by α-(1,4) glucosidic bonds, with lipophilic inner cavities and hydrophilic outer surfaces. They are able to entrap hydrophobic drugs in their cavities, forming non-covalent inclusion complexes, thus allowing the dissolution in the aqueous phase of the drug included, making it suitable to diffuse in an aqueous medium, to come in contact with the membrane surface, and to permeate through the membrane. CDs are also able to interact with membrane components and to solubilize cholesterol, inducing perturbation in the lipid bilayer, and affect the membrane properties, such as fluidity and permeability. Moreover, the encapsulation in CDs protects the drug from chemical and enzymatic degradation [19,20,21,22].

Here, inclusion complexes of both PTA34 and PTA73 in hydroxy-propil-β-cyclodextrin (HP-β-CD), a semisynthetic cyclodextrin approved by the Food and Drug Administration (FDA) as an excipient for parenteral formulations, were developed (Figure 1) [23]. In detail, the inclusion complexes among the HP-β-CD and these biphenylnicotinamide derivatives were studied first in solution, by the analysis of the phase solubility diagram, according to Higuchi–Connors [24], and the construction of the Job plot [18] for the identification of the host–guest stoichiometric ratio. Next, the inclusion complexes were prepared at the solid state by freeze-drying and characterized in terms of incorporation degree and dissolution profiles.

Finally, in order to evaluate the effectiveness of the PTA’s complexation in HP-β-CD, in terms of antitumor activity improvement, cytotoxicity studies, cell cycle analysis, and apoptosis determination were conducted in a panel of PDAC cell lines, AsPC-1, PANC-1, and MIA PaCa-2. The activities of the complexes PTA34/HP-β-CD and PTA73/HP-β-CD were compared to those of the corresponding pure molecules, showing a higher antiproliferative efficacy and an unaltered activity in terms of modulation of the cell cycle.

In conclusion, the two new cyclodextrin inclusion complexes have proven in vitro to be promising candidates for PDAC therapy, even if in vivo studies are needed in order to validate an actual clinical use of these formulations.

## 2. Results and Discussion

### 2.1. Solubility and Phase-Solubility Studies of PTA73 and PTA34

**Figure 2:**
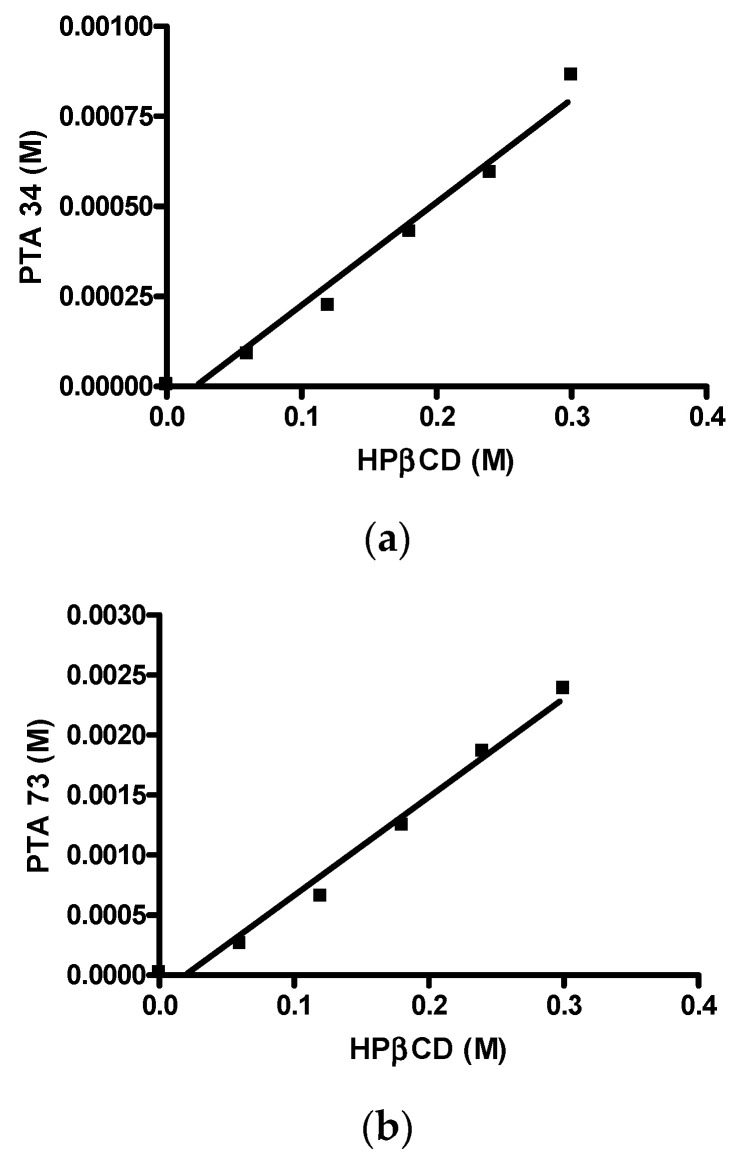
Phase solubility diagrams. (**a**) PTA34/HP-β-CD; (**b**) PTA73/HP-β-CD.

First of all, the solubility of PTA73 and PTA34 was determined at 37 ± 0.5 °C in ultra-pure water, and the results showed that the solubility is critical for the bioavailability of these compounds. In detail, the intrinsic solubility (S_0_), was equal to 1.95 µg/mL (5.78 × 10^−6^ M) and 7.16 µg/mL (2.24 × 10^−5^ M) for PTA 34 and PTA 73, respectively, thus complexation with a cyclodextrin could represent a valid solubilization strategy for these compounds. In Figure 2a,b the phase solubility diagrams of both the compounds are reported. It is evident that there is a linear correlation between solubility and HP-β-CD concentration, therefore both diagrams are of the A_L_ type, according to the classification proposed by Higuchi–Connors [24]. The solubility values obtained at 37 ± 0.5 °C in the presence of different HP-β-CD concentrations are shown in Table 1. The linear trend of the Higuchi–Connors diagram certifies the formation of an inclusion complex, with 1:1 host:guest stoichiometry, so that using the relative equation, as reported in the materials and methods section, it was possible to calculate the relative equilibrium constant (Ks). In particular, for PTA34 the presence of HP-β-CD at a maximum concentration of 45% *w/v* led to an increase in terms of aqueous solubility of about 150 times, bringing it from an initial solubility value of 1.95 µg/mL to a final solubility value of 292.5 µg/mL, and the calculated equilibrium constant was 503.9 M^−1^. For PTA73, instead, the presence of HP-β-CD at a maximum concentration of 45% *w/v* led to an increase in terms of aqueous solubility of about 106 times, bringing it from an initial solubility value of 7.16 µg/mL to a final solubility value of 762.5 µg/mL, and the calculated equilibrium constant was 369.2 M^−1^.

The calculated Ks values indicated a good complexing capacity of the HP-β-CD towards these two new N-biphenylnicotinamide derivatives. Furthermore, PTA34 presented a higher complexation constant value, and this result is explained by the presence on aromatic ring of the para fluorine atom. Since the fluorine atom is a lipophilic substituent, PTA34 also showed a lower water solubility.

### 2.2. Job’s Plot Method

**Figure 3:**
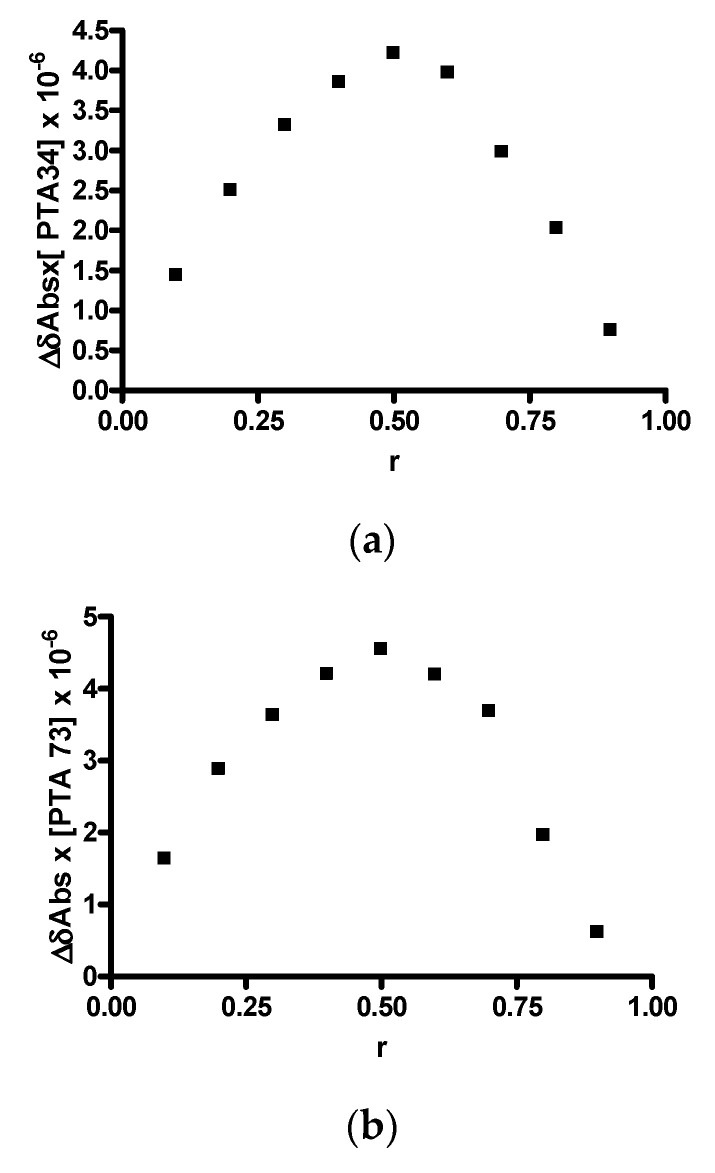
Job plots. (**a**) PTA 34/HP-β-CD; (**b**) PTA 73/HP-β-CD.

In order to investigate the host-guest stoichiometric ratio and to confirm the linear behavior of the Higuchi–Connors diagrams, the construction of the Job plot was carried out. Since both compounds under analysis exhibited absorption in the visible spectrum, this determination was conducted via UV-VIS spectrophotometry and the graphs obtained are shown in Figure 3a,b. In both cases, a highly symmetrical trend, with a maximum value recorded at *r* = 0.5, is observed, highlighting the formation of a 1:1 inclusion complex. This result is fully in agreement with the A_L_ trend of the solubility diagrams.

### 2.3. Preparation of PTA34 or PTA73/HP-β-CD Inclusion Complexes at the Solid State and Determination of Their Incorporation Degree

The inclusion complexes PTA34/HP-β-CD and PTA73/HP-β-CD were also prepared in the solid state by freeze-drying. A solid-state drug-cyclodextrin inclusion complex is certainly the most suitable tool to allow the administration of the drug, both orally and parenterally, overcoming the limit represented by its low solubility in water [17]. The lyophilized complexes were characterized through the determination of the incorporation degree, expressed as mg of PTA34 or PTA73 per 1 g of product, and were found to be 1.23 ± 0.42 and 2.91 ± 1.0 mg of drug per 1 g of lyophilized powder for PTA34 or PTA73, respectively.

### 2.4. In Vitro Dissolution Studies

**Figure 4:**
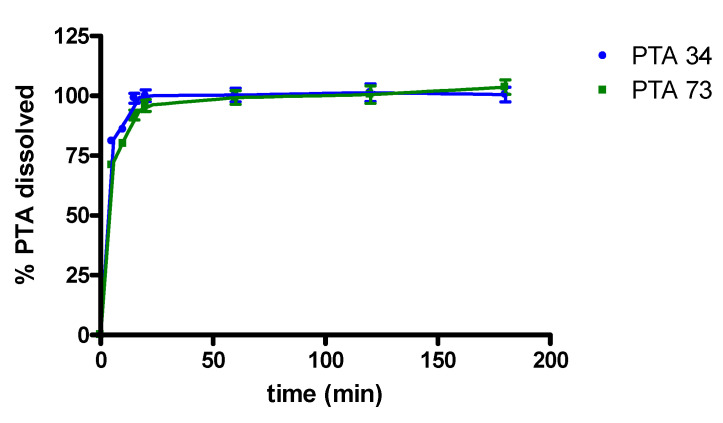
Dissolution profiles of PTA/HP-β-CD solid complexes.

In Figure 4 in vitro dissolution profiles, in 0.05 M phosphate buffer at pH 7.4, of PTA34 and PTA73, from their respective solid-state inclusion complexes, are shown. In the same graph the dissolution profiles of the two uncomplexed compounds are not reported because, due to their very low solubility, the dissolved quantity was well below the detection limit, and this prevented the quantitative determination via UV-VIS spectrophotometry in the dissolution medium. The lipophilic nature of both drugs limits their contact with the dissolution medium, causing them to float on the surface and hindering their dissolution. On the contrary, the freeze-dried complexes dissolve very quickly once they are placed in the dissolution medium and, in both cases, 100% of the complexed drug was solubilized within the first 20 min of the dissolution process.

Consequently, the complexation with HP-β-CD certainly represents a valid strategy for improving the solubility characteristics and the dissolution profile of these two biphenylnicotinamide derivatives.

### 2.5. In Vitro Studies

#### 2.5.1. Cytotoxicity

**Figure 5:**
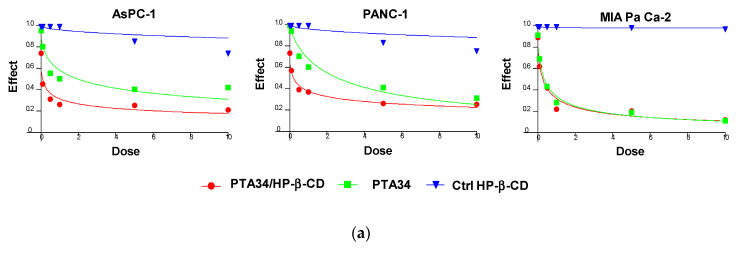

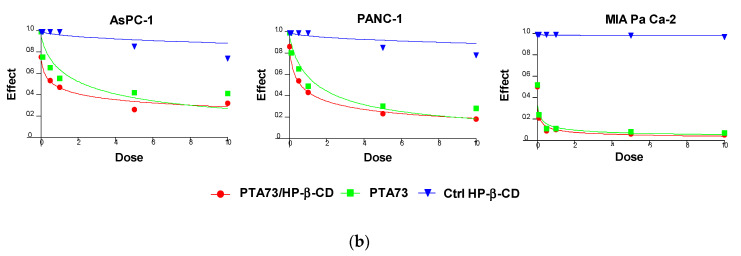
Dose/effect plots of the mean of three different cell proliferation experiments, conducted in PDAC cell lines incubated for 72 h with HP-β-CD (Ctrl HP-β-CD), PTA34, and PTA34/HP-β-CD (panel (**a**)) or with HP-β-CD (Ctrl HP-β-CD, PTA73, and PTA73/HP-β-CD (Panel (**b**)). Dose was expressed in each graph as 0.01–10 µM concentration range, in terms of PTA34 or PTA73, corresponding to 2.9–2900 µg/mL concentration range for HP-β-CD alone.

The effectiveness of PTA34 and PTA73, both complexed in HP-β-CD and as pure compounds, was evaluated in three human pancreatic cancer cell lines AsPC-1, Panc-1, and MIA PaCa-2 cells, by MTT assay after 72 h of treatment. In order to demonstrate that the complexed drugs (PTA 34/HP-β-CD and PTA 73/HP-β-CD) did not lose their antiproliferative activity against tumor cells, in respect to uncomplexed ones (PTA34 and PTA73, respectively), the proliferation of all cells was determined after each drug exposure, and IC_50_ was calculated. The dose/effect curves of PTA34/HP-β-CD vs. PTA34 (Figure 5, Panel (a)) and of PTA73/HP-β-CD vs. PTA73 (Figure 5, Panel (b)), as well as the IC_50_ values reported in Table 2, show that both the complexes were even more active than the non-complexed ones, in AsPC- 1 and in PANC-1 cells, while in MIA PaCa-2 cells, where the lowest IC_50_ values for PTA34 and PTA73 alone were recorded, the activity of the complexed compounds and pure molecules was comparable.

Furthermore, to exclude that the higher cytotoxic activity of the complexed compounds was to some extent attributable to the HP-β-CD, this was tested in the same concentration ranges utilized in the drug complexes. The dose/effect curves in Figure 5 for cyclodextrin (Ctrl HP-β-CD) clearly show that it was not toxic at the analyzed concentration range. These results evidenced a general improvement in the cytotoxic activity of these drugs following complexation, due to the increase in solubility in aqueous medium and to the enhancement of the plasmatic membrane permeability by HP-β-CD. As reported in the literature, this FDA approved excipient is characterized by a hydrophobic pocket capable of binding and solubilizing cholesterol, a critical component of the plasma membrane, thus having a role in the efflux and redistribution of cholesterol in mammalian cells [23,25,26,27]. Thus, the interaction of HP-β-CD with cholesterol in the plasma membrane can induce perturbation in the lipid bilayer, affecting the membrane properties such as fluidity and permeability. For these reasons, it is plausible to speculate that the cyclodextrin in the formulation of PTA compounds triggered the increase of intracellular uptake of the complexed drugs by enhancing the plasma membrane permeability. Ultimately, HP-β-CD could be considered a drug delivery enhancer for PTA 34 and PTA73.

#### 2.5.2. Cell Cycle Modulation

**Figure 6:**
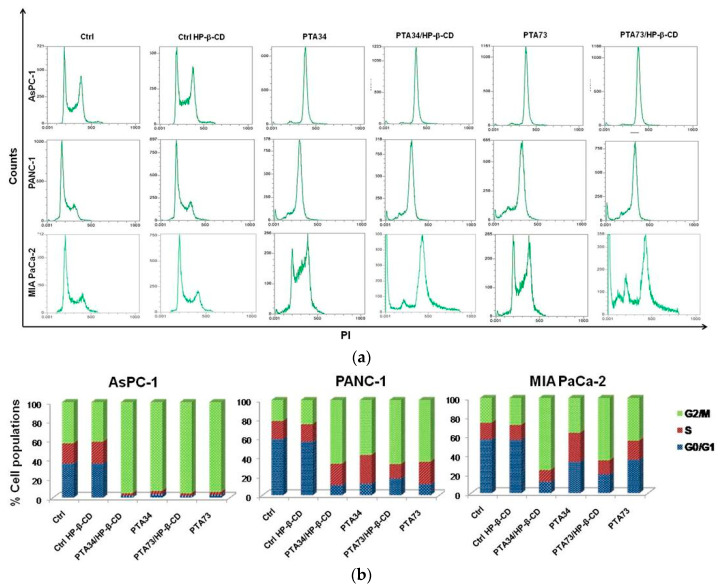
Effects on cell cycle of PDAC cells by CD (Ctrl-CD), complexed PTA34 and PTA73 (PTA34/HP-β-CD and PTA73/HP-β-CD, respectively) and PTA34 and PTA73, alone. The modulation of cell cycle phases was evaluated by flow cytometry (FCM) analysis after staining cells with propidium iodide. In panel (**a**), a representative analysis of three independent experiments is reported for all cell lines investigated. In panel (**b**) the bar graph shows cell population percentage in each phase of the cell cycle.

In order to investigate whether the PTA’s complexation in HP-β-CD altered their mechanism of action, in terms of cell cycle modulation compared to uncomplexed drugs, we conducted the flow cytometry (FCM) analysis, after staining with propidium iodide, of cells previously treated for 24 h with compounds at 1µM in terms of PTA’s concentration. In Figure 6, representative FCM histograms of cell cycle modulation by complexed drugs, or pure compounds in the three cell lines, are reported, as well as the quantification of three independent experiments. Results showed a strong arrest in the G2/M phase of AsPC-1 cells, induced by complexed and uncomplexed PTAs, with 93% vs. 95.50% and 94.1% vs. 95.58% for PTA34 vs. PTA34/HP-β-CD and PTA73 vs. PTA73/HP-β-CD, respectively. PANC-1 and MIA PaCa-2 cells were found to be less responsive to drugs with 57.9% vs. 66.9% and 64.9% vs. 67.3% for PTA34 vs. PTA34/HP-β-CD and PTA73 vs. PTA73/HP-β-CD, respectively in PANC-1 cells, and 36.7% vs..75.74% and 45.2% vs. 65.7% for PTA34 vs. PTA34/HP-β-CD and PTA73 vs. PTA73/HP-β-CD, respectively, in MIA PaCa-2 cells. However, in PANC-1 and MIA PaCa-2 cells, the complexed drugs induced an increase in the percentage of cells arrested in G2/M phase, compared to uncomplexed drugs, confirming an improvement of the drugs activity. In conclusion, PTA34/HP-β-CD resulted more active than PTA34 in inducing the arrest in G2/M phase in all PDAC cell lines while PTA73/HP-β-CD resulted more active than its pure counterpart only in MIA PaCa2 cells. Moreover, HP-β-CD alone (Ctrl HP-β-CD), tested at a concentration of 290 µg/mL, did not alter the cell cycle in all three investigated cell lines. These results allowed us to confirm that the complexation of these two biphenylnicotinamide derivatives with HP-β-CD, per se, did not modify the blocking activity in the G2/M phase of the cell cycle but may in some cases increase its effectiveness.

#### 2.5.3. Apoptosis Assay

**Figure 7:**
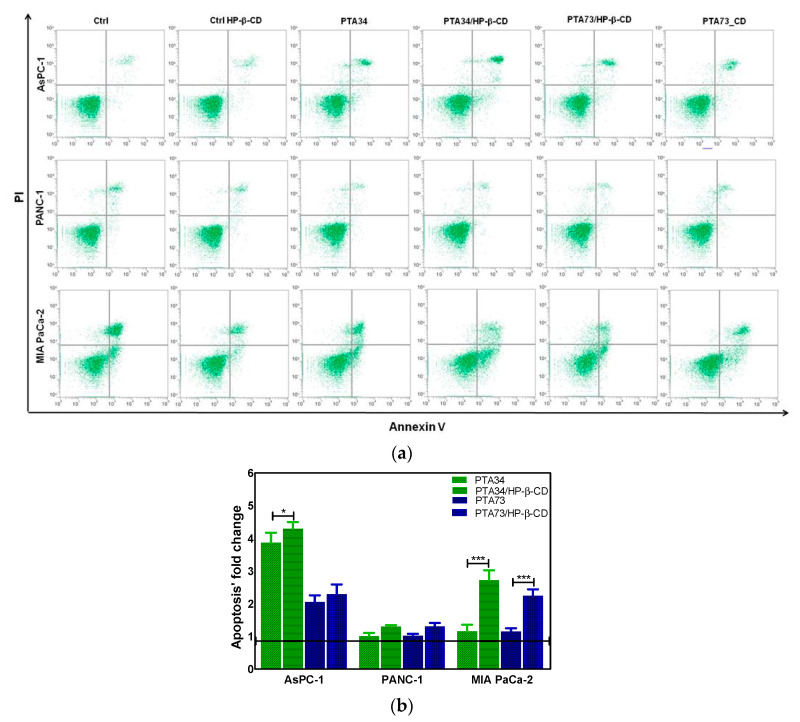
(**a**) Representative apoptosis analysis of PDAC cells after 24 h treatment with HP-β-CD alone (Ctrl HP-β-CD), PTA34, PTA34/HP-β-CD, PTA73, and PTA73/HP-β-CD. Apoptosis was evaluated by using Annexin V/PI staining followed by FCM analysis. (**b**) Fold change of Annexin V positive cells (early plus late apoptosis) in treated samples versus their corresponding reference compounds (Ctrl cells and Ctrl HP-β-CD for PTAs and complexed-PTAs treated cells, respectively). The results are the mean ± SD of three independent experiments (* *p* < 0.05; *** *p* < 0.001).

The results of cell cycle analysis prompted us to investigate if the observed phase arrest in G2/M was followed by induction of apoptosis. Thus, the FITC Annexin V staining was carried out after 24 h of incubation of PDAC cells with PTA34 and PTA73 free and complexed in HP-β-CD at the same concentration used for cell cycle analysis (1 µM in terms of PTA’s). Untreated cells and HP-β-CD treated ones, were used as references for PTAs and PTA’s/HP-β-CD complexes, respectively. As representative of apoptosis analysis, the FCM dot plots of Annexin V/PI positive PDAC cells are reported in Figure 7a, whereas fold changes of Annexin V positive cells (early apoptosis plus late apoptosis) in treated samples, versus their specific reference compound, are summarized in Figure 7b. The induction of apoptosis was more evident in AsPC-1 cells when treated with PTA34 complexed and not in respect to PTA73 and PTA73/HP-β-CD, conversely, in MIA PaCa-2 cells both the complexes showed a higher effectiveness than the uncomplexed counterpart. PANC-1 cells showed a completely different behavior, the arrest in G2/M phase after 24 h of treatment did not trigger the marked induction of apoptosis, rather, only a slowdown in cell growth, as observed in cell viability assay. Finally, the HP-β-cyclodextrin alone did not affect the basal condition of cells in terms of apoptotic events (Figure 7a).

A possible explanation for the different sensitivity to these drugs, pure or complexed, of PDAC cell lines may lie in their cellular cholesterol levels. Li et al. documented an aberrant accumulation of cholesteryl ester (CEs) in human pancreatic cancer tissues and cell lines, but not in normal counterparts and, specifically, MIA PaCa-2 and PANC-1 cells had much higher levels of CEs than AsPC-1 [28]. Briefly, mammalian cells obtain cholesterol either from de novo synthesis or from the uptake of low-density lipoprotein (LDL). Overaccumulation of free (unesterified) cholesterol can be toxic to cells, thus to prevent accumulation, cholesterol is converted primarily by the enzyme ACAT1, to CEs, which mainly exists as cytosolic lipid droplets. A different enzyme, the cholesteryl ester hydrolase (CEH), acts in the opposite direction by liberating cholesterol from CEs, which now can reach the plasma membrane. Biosynthesis and hydrolysis of CEs occur continuously, forming the cholesterol/CE cycle [29]. Thus, given the existence of the free cholesterol/esterified cholesterol cycle, it is plausible to hypothesize that low levels of esterified intracellular cholesterol correspond to low levels of cholesterol in the plasma membrane and vice versa. This might explain why AsPC-1 cells, having the lowest esterified-cholesterol levels compared to the other two PDAC cell lines, are more sensitive to the cyclodextrin-dependent solubilizing action of cholesterol, which in turn results in both increased intracellular uptake and antitumor activity of the delivered PTAs.

## 3. Materials and Methods

### 3.1. Materials

The synthesis of PTA34 (MW = 337.30) and PTA73 (MW = 319.30) was performed according to the already reported literature procedure [6,7]. HP-β-CD (hydroxypropil-β-cyclodextrin, MW = 1396 Dalton, substitution degree 0.65) was purchased from Farmalabor (Canosa, Italy). HCl and phosphate salts for the preparation of buffers were purchased from Fluka (Sigma Aldrich, Milan, Italy). Bidistilled water was bought from Carlo Erba (Milan, Italy). All other products and reagents used in this work were of analytical grade. Pancreatic cancer cell lines PANC-1, AsPC-1 and MIAPaCa-2 cells were purchased from ATCC. The MTT used for cytotoxicity studies and PI for cell cycle analysis were purchased from Sigma Aldrich (Milan, Italy). FITC Annexin V Apoptosis Detection Kit I was from BD PharmingenTM (San Jose, CA, USA, 556547).

### 3.2. Quantitative Analysis of PTA34 and PTA73

Quantitative analysis of PTA34 and PTA73 was carried out by spectrophotometry using a dual-beam UV-VIS Lambda 20 Bio spectrophotometer (Perkin Elmer, Milan, Italy) and quartz cuvettes, with a capacity of 1 mL, using methanol as solvent. In the case of PTA34 the reading was carried out at a wavelength of 320 nm, and a calibration straight line (r^2^ = 0.9979), in the concentration range between 0.15 mg/mL (4.45 × 10^−4^ mM) and 0.015 mg/mL (4.45 × 10^−5^ mM), was obtained. For PTA73 the reading was carried out at a wavelength of 340 nm, and a calibration straight line (r^2^ = 0.9999) in the concentration range between 0.16 mg/mL (5.01 × 10^−4^ mM) and 0.016 mg/mL (5.01 × 10^−5^ mM), was obtained.

### 3.3. Solubility and Phase-Solubility Studies of PTA34 and PTA73

The phase solubility study was conducted according with Higuchi and Connors. In detail, an excess of PTA34 or PTA73 was added to 1 mL of solutions containing HP-β-CD in the appropriate concentration (0–45% *w/v*) in 4 mL vials, with screw caps to avoid evaporation. The obtained mixtures were vigorously vortexed for about 5 min, and the suspensions thus obtained were placed under constant stirring in a thermostat bath at 37 ± 0.5 °C for about 48 h, until the saturation of the system was achieved. Then, each suspension was centrifuged at 13,200 rpm (MIKRO 22 R, Hettich Zentrifugen, Germany) in order to remove the uncomplexed drug and the supernatant was analyzed by spectrophotometry for the quantification of the drug, after appropriate dilution. All determinations were conducted at least in triplicate. The obtained data were used to determine the apparent stability 1:1 constant (K_1:1_) of the HP-β-CD inclusion complexes with the biphenylnicotinamide derivatives, using the Higuchi and Connors equation:

$$K_{1:1}=\frac{slope}{S_{0}(1-slope)}$$

where *slope* is the slope of the obtained phase solubility diagrams straight line, and *S*_0_ represents PTA34 or PTA73 intrinsic solubility determined in the same way.

### 3.4. Job’s Plot Method

The stoichiometric ratio between PTA34 or PTA73 and HP-β-CD in the inclusion complexes was determined by the continuous variation method or Job’s plot method [18]. In detail, starting from CH_3_OH/H_2_O (55/45 *v/v*) equimolar solutions (1.0 × 10^−4^ M) of PTA34/PTA73 and HP-β-CD, and keeping the total molar concentration of the species constant, a set of intermediate solutions with fixed volume was obtained by mixing them in the molar ratio ranged from 0 to 1. After stirring for 1 h, for each solution the absorbance (abs) was registered by UV–VIS spectroscopy at 320 nm for PTA34, and at 340 nm for PTA73. Then, ΔAbs × [PTA34 or PTA73] was plotted versus r, where

$$r=\frac{\left[PTA34orPTA73\right]}{\left[PTA34orPTA73\right]+\left[HP-\beta -CD\right]}$$

### 3.5. Preparation of PTA34 or PTA73/HP-β-CD Inclusion Complexes at the Solid State and Determination of Their Incorporation Degree

The PTA34 or PTA73 and HP-β-CD (PTA34/HP-β-CD or PTA73/HP-β-CD) inclusion complex was prepared in the solid state by freeze drying. Briefly, PTA34 or PTA73 and HP-β-CD were placed in water in a 1:1 molar ratio and the suspension was at first vigorously vortexed for about 5 min and then left under stirring for two days. At the end of the two days the samples were filtered through 0.22 µM cellulose acetate filters (Millipore), frozen, and freeze-dried (Lio 5P, Milan, Italy). The amount of PTA34 or PTA73 present in the PTA34 or PTA73/HP-β-CD solid complex was determined by solubilizing about 5 mg of each sample in 5 mL of deionized water. The obtained solution was filtered with 0.22 µM cellulose acetate filters (Millipore^®^), and the quantification of the drug was carried out via UV-VIS spectrophotometry, as described in Section 3.2.

The incorporation degree of PTA34 or PTA73 into the inclusion complexes was determined from the obtained absorbance, using the calibration straight line, and expressed as mg of PTA34 or PTA73 per 1 g of complex.

### 3.6. In Vitro Dissolution Studies

Dissolution experiments were performed at 37 °C according to the paddle method [30] and maintaining a rotational speed of 100 rpm during the test. Suitable quantities of PTAs/HP-β-CD solid complexes were suspended in the dissolution medium (0.05 M phosphate buffer at pH 7.4). At predetermined time intervals, 600 µL of suspension was collected and, in order to keep constant the initial volume, 600 µL of the same dissolution medium previously thermostated at the same temperature was added. Samples were subsequently filtered using a 0.22 µm membrane filter (Millipore^®^ cellulose acetate), and the filtrates thus obtained were subjected to UV-VIS analysis after appropriate dilution. For quantitative analysis the calibration curve previously constructed was used, and the dissolution profiles shown correspond to the average of three determinations.

### 3.7. Cell Culture

Human pancreatic ductal adenocarcinoma (PDAC) PANC-1 cells, human pancreas adenocarcinoma ascites metastasis cells AsPC-1, and undifferentiated human pancreatic carcinoma MIAPaCa-2 cells, were purchased from ATCC^®^. PANC-1 and AsPC-1 cells were cultured in Dulbecco’s modified Eagle’s medium (DMEM), supplemented with fetal bovine serum (FBS), to a final concentration of 10%, L-glutamine 1% (*v/v*), and penicillin/streptomycin 1% (*v/v*). MIA PaCa-2 cells were grown in Roswell Park Memorial Institute (RPMI) medium, supplemented as above, and in addition with horse serum, to a final concentration of 2.5%. Cells were maintained in a humid atmosphere of 95% air and 5% CO_2_ at 37 °C. All materials for cell culturing were purchased from EuroClone (Pero, Milan, Italy).

### 3.8. Cell Proliferation Assay

The 3-(4,5-dimethylthiazol-2-yl)-2,5- diphenyltetrazoliumbromide (MTT) assay was performed as previously described [5] to investigate the effect of hydroxy-propil-β-cyclodextrin-complexed PTA34 and PTA73 (PTA34/HP-β-CD and PTA73/HP-β-CD, respectively) on cell viability of PANC-1, AsPC-1, and MIA PaCa-2 cell lines. The same uncomplexed compounds and HP-β-CD alone were also tested as reference compounds. Untreated cells were used as a positive control. In particular, cells were seeded in 96 well plates at a density of 5000 cells/well, incubated for 24 h in culturing medium, and subsequently treated for 72 h, with compounds at 0.01–10 µM concentrations range, in terms of PTA34 or PTA73, included. HP-β-CD–cyclodextrin alone was tested in the concentrations range corresponding to that present in the complex, namely 2.9–2900 µg/mL. After the incubation period 10 µL of a 0.5% (*w/v*) MTT/PBS solution were added to each well, and the incubation was prolonged further for 2 h. After this period, medium was removed and replaced with 100 µL of DMSO. The absorbance in each well was measured by a microplate reader (MULTISKAN Sky, ThermoScientific). The results are shown as dose/effect plots of the mean of three different experiments. The IC_50_ was defined as the drug concentration yielding a fraction of affected (no surviving) cells = 0.5, normalized with untreated cells, and was calculated utilizing CalcuSyn v.1.1.1 software (Biosoft, UK).

### 3.9. Cell Cycle Analysis

For the cell cycle analysis, human pancreatic cancer cells were seeded in 60 mm dishes at a density of 500,000/well and incubated for 24 h at 37 °C, with PTA34, PTA73 both complexed and not at concentration 1 µM in terms of PTA’s, and with cyclodextrin alone at concentration equal to 290 µg/mL. Cells were then harvested, washed twice in ice-cold PBS pH 7.4, fixed in 4 mL of 70% ethanol, and stored at -20 °C until analysis. The cell cycle modulation induced by treatments was studied as previously described [7] by propidium iodide (PI) staining; the pellet was resuspended in PBS without Ca^2+^ and Mg^2+^, containing 1 mg/mL RNase, 0.01% NP40, and 50 ĉg/mL PI (Sigma), and then flow cytometry analysis was performed on Attune NxT acoustic focusing cytometer (Life Technologies). Data were interpreted using the CytExpert software v.1.2, provided by the manufacturer.

### 3.10. Annexin V/PI Apoptosis Assay

Annexin V/PI assay was conducted as previously described [31] to understand the mechanism of cell death, occurring after cell cycle arrest in phase G2/M. In particular after 24 h of treatment with compounds at 1 µM in terms of PTA’s concentration, cells were subjected to Annexin-V FITC/propidium iodide staining, which allowed detection of early and late apoptosis as Annexin V positive cells, and late apoptosis as Annexin V/PI positive cells, and summarized in a graph as fold change of Annexin V positive cells (Annexin V plus Annexin V/PI) in treated samples versus their specific reference compound. FITC Annexin V Apoptosis Detection Kit I was from BD PharmingenTM (San Jose, CA, USA, 556547) and the analysis was performed on Attune NxT acoustic focusing cytometer (Life Technologies). Data were interpreted using the CytExpert software v. 1.2 provided by the manufacturer.

## 4. Conclusions

In conclusion, the complexation of PTA34 and PTA73 with hydroxy-propil-β-cyclodextrin was successful both in solution and in solid state, allowing an increase of the PTA’s water solubility and a favorable dissolution profile with respect to the uncomplexed drug. In addition, the property of hydroxy-propil-β-cyclodextrin, which allows the enhancement of the plasma membrane permeability, was responsible for the increase of intracellular uptake of the complexed drugs, and consequently of their antitumor efficacy, as evidenced in studies conducted on PDAC cells model. Considering the promising results obtained, and that hydroxy-propil-β-cyclodextrin is an excipient already approved by the FDA for parenteral formulations, the inclusion complexes of PTA34 and PTA73, after an in vivo validation step, could be considered for parenteral administration in PDAC therapy.

## Figures and Tables

**Table 1:** PTA34 and PTA73 Water Solubility Values in Presence of HP-β-CD.

HP-β-CD (% *w/v*)	PTA34 (µg/mL)	PTA73 (µg/mL)
**0**	1.95 ± 0.35	7.16 ± 0.34
**9**	30.4 ± 4.3	84.1 ± 4.1
**18**	76.1 ± 3.9	209.5 ± 9.2
**27**	145.0 ± 7.1	398.4 ± 19.1
**36**	200.2 ± 28.3	596.0 ± 48.1
**45**	292.5 ± 24.7	762.5 ± 137.9

Data are the means of at least three determinations.

**Table 2:** Inhibitory Effect of PTA Compounds and PTA/HP-β-CD Complexes on Pancreatic Ductal Adenocarcinoma (PDAC) Cancer Cells.

	IC_50_ (µM ± SD)			
Cell Lines	PTA34/HP-β-CD	PTA34	PTA73/HP-β-CD	PTA73
**AsPC-1**	0.09 ± 0.02	1.9 ± 0.3	0.66 ± 0.20	2.3 ± 0.6
**Panc-1**	0.20 ± 0.02	2.5 ± 0.5	0.62 ± 0.02	1.5 ± 0.2
**MIA PaCa-2**	0.27 ± 0.05	0.31 ± 0.04	0.005 ± 0.001	0.006 ± 0.002

## Abbreviations

PDAC	Pancreatic ductal adenocarcinoma
GEM	Gemcitabine
NAB	Nabpaclitaxel
HP-β-CD	Hydroxy-Propil-β-Cyclodextrin
CDs	Cyclodextrins
MTAs	Microtubule targeting agents
FDA	Food and Drug Administration
FACS	Fluorescence-Activated Cell Sorter

## References

[1] American Cancer Society Journal (2020). Cancer Facts & Figures 2020. CA Cancer J. Clin..

[2] Associazione Italiana di Oncologia Medica (AIOM) (2019). Linee Guida: Carcinoma del Pancreas Esocrino. Aiom.

[3] Chen X., Zhou W., Liang C., Shi S., Yu X., Chen Q., Sun T., Lu Y., Zhang Y., Guo Q. (2019). Codelivery Nanosystem Targeting the Deep Microenvironment of Pancreatic Cancer. Nano Lett..

[4] Veenstra V., Garcia-Garijo A., van Laarhoven H., Bijlsma M. (2018). Extracellular Influences: Molecular Subclasses and the Microenvironment in Pancreatic Cancer. Cancers (Basel).

[5] Porcelli L., Iacobazzi R., Di Fonte R., Serratì S., Intini A., Solimando A., Brunetti O., Calabrese A., Leonetti F., Azzariti A. (2019). CAFs and TGF-β Signaling Activation by Mast Cells Contribute to Resistance to Gemcitabine/Nabpaclitaxel in Pancreatic Cancer. Cancers (Basel).

[6] Majellaro M., Stefanachi A., Tardia P., Vicenti C., Boccarelli A., Pannunzio A., Campanella F., Coluccia M., Denora N., Leonetti F. (2017). Investigating Structural Requirements for the Antiproliferative Activity of Biphenyl Nicotinamides. ChemMedChem.

[7] Porcelli L., Stolfa D., Stefanachi A., Di Fonte R., Garofoli M., Iacobazzi R.M., Silvestris N., Guarini A., Cellamare S., Azzariti A. (2019). Synthesis and biological evaluation of N-biphenyl-nicotinic based moiety compounds: A new class of antimitotic agents for the treatment of Hodgkin Lymphoma. Cancer Lett..

[8] Gagliano N., Volpari T., Clerici M., Pettinari L., Barajon I., Portinaro N., Colombo G., Milzani A., Dalle-Donne I., Martinelli C. (2012). Pancreatic cancer cells retain the epithelial-related phenotype and modify mitotic spindle microtubules after the administration of ukrain in vitro. Anticancer Drugs.

[9] Chan K.-S., Koh C.-G., Li H.-Y. (2012). Mitosis-targeted anti-cancer therapies: Where they stand. Cell Death Dis..

[10] Vila-Navarro E., Fernandez-Castañer E., Rovira-Rigau M., Raimondi G., Vila-Casadesus M., Lozano J.J., Soubeyran P., Iovanna J., Castells A., Fillat C. (2020). MiR-93 is related to poor prognosis in pancreatic cancer and promotes tumor progression by targeting microtubule dynamics. Oncogenesis.

[11] Kumar S., Parkash C., Kumar P., Singh S.K. (2009). Application of some novel techniques for solubility enhancement of mefenamic acid, a poorly water soluble drug. Int. J. Pharm. Sci. Drug Res..

[12] Dora C.P., Singh S.K., Kumar S., Datusalia A.K., Deep A. (2010). Development and characterization of nanoparticles of glibenclamide by solvent displacement method. Acta Pol. Pharm.—Drug Res..

[13] Tang C., Guan Y.-X., Yao S.-J., Zhu Z.-Q. (2014). Solubility of Dexamethasone in Supercritical Carbon Dioxide with and without a Cosolvent. J. Chem. Eng. Data.

[14] Iacobazzi R.M., Letizia P., Assunta L.A., Valentino L., Antonio L., Annalisa C., Emiliano A., Roberta D.F., Amalia A., Massimo F. (2017). Targeting human liver cancer cells with lactobionic acid-G(4)-PAMAM-FITC sorafenib loaded dendrimers. Int. J. Pharm..

[15] Lopedota A., Cutrignelli A., Laquintana V., Denora N., Iacobazzi R.M., Perrone M., Fanizza E., Mastrodonato M., Mentino D., Lopalco A. (2016). Spray Dried Chitosan Microparticles for Intravesical Delivery of Celecoxib: Preparation and Characterization. Pharm. Res..

[16] Cutrignelli A., Sanarica F., Lopalco A., Lopedota A., Laquintana V., Franco M., Boccanegra B., Mantuano P., De Luca A., Denora N. (2019). Dasatinib/HP-β-CD inclusion complex based aqueous formulation as a promising tool for the treatment of paediatric neuromuscular disorders. Int. J. Mol. Sci..

[17] Devasari N., Dora C.P., Singh C., Paidi S.R., Kumar V., Sobhia M.E., Suresh S. (2015). Inclusion complex of erlotinib with sulfobutyl ether-β-cyclodextrin: Preparation, characterization, in silico, in vitro and in vivo evaluation. Carbohydr. Polym..

[18] Cutrignelli A., Lopedota A., Denora N., Iacobazzi R.M., Fanizza E., Laquintana V., Perrone M., Maggi V., Franco M. (2014). A new complex of curcumin with sulfobutylether-β-cyclodextrin: Characterization studies and in vitro evaluation of cytotoxic and antioxidant activity on HepG-2 cells. J. Pharm. Sci..

[19] Challa R., Ahuja A., Ali J., Khar R.K. (2005). Cyclodextrins in drug delivery: An updated review. AAPS PharmSciTech.

[20] Másson M., Loftsson T., Másson G., Stefánsson E. (1999). Cyclodextrins as permeation enhancers: Some theoretical evaluations and in vitro testing. J. Control. Release.

[21] Hammoud Z., Khreich N., Auezova L., Fourmentin S., Elaissari A., Greige-Gerges H. (2019). Cyclodextrin-membrane interaction in drug delivery and membrane structure maintenance. Int. J. Pharm..

[22] Rong W.-T., Lu Y.-P., Tao Q., Guo M., Lu Y., Ren Y., Yu S.-Q. (2014). Hydroxypropyl-Sulfobutyl-β-Cyclodextrin Improves the Oral Bioavailability of Edaravone by Modulating Drug Efflux Pump of Enterocytes. J. Pharm. Sci..

[23] Szente L., Szejtli J. (1999). Highly soluble cyclodextrin derivatives: Chemistry, properties, and trends in development. Adv. Drug Deliv. Rev..

[24] Higuchi T., Connors K.A. (1965). Techniques, Phase solubility. Adv. Anal. Chem. Instrum..

[25] Silvente-Poirot S., Poirot M. (2014). Cholesterol and Cancer, in the Balance. Science.

[26] Brown A., Patel S., Ward C., Lorenz A., Ortiz M., DuRoss A., Wieghardt F., Esch A., Otten E.G., Heiser L.M. (2016). PEG-lipid micelles enable cholesterol efflux in Niemann-Pick Type C1 disease-based lysosomal storage disorder. Sci. Rep..

[27] Kilsdonk E.P.C., Yancey P.G., Stoudt G.W., Bangerter F.W., Johnson W.J., Phillips M.C., Rothblat G.H. (1995). Cellular cholesterol efflux mediated by cyclodextrins. J. Biol. Chem..

[28] Li J., Gu D., Lee S.S.-Y., Song B., Bandyopadhyay S., Chen S., Konieczny S.F., Ratliff T.L., Liu X., Xie J. (2016). Abrogating cholesterol esterification suppresses growth and metastasis of pancreatic cancer. Oncogene.

[29] Chang T.-Y., Chang C.C.Y., Ohgami N., Yamauchi Y. (2006). Cholesterol Sensing, Trafficking, and Esterification. Annu. Rev. Cell Dev. Biol..

[30] Cutrignelli A., Lopedota A., Trapani A., Boghetich G., Franco M., Denora N., Laquintana V., Trapani G. (2008). Relationship between dissolution efficiency of Oxazepam/carrier blends and drug and carrier molecular descriptors using multivariate regression analysis. Int. J. Pharm..

[31] Azzariti A., Iacobazzi R.M., Di Fonte R., Porcelli L., Gristina R., Favia P., Fracassi F., Trizio I., Silvestris N., Guida G. (2019). Plasma-activated medium triggers cell death and the presentation of immune activating danger signals in melanoma and pancreatic cancer cells. Sci. Rep..

